# Role of Neuromodulators for the Management of Post-Gastric-Fundoplication Dyspepsia: A Retrospective Series

**DOI:** 10.7759/cureus.18343

**Published:** 2021-09-28

**Authors:** Achintya Singh, Andrew M Ford, John McMichael, Scott Gabbard

**Affiliations:** 1 Internal Medicine, Cleveland Clinic Foundation, Cleveland, USA; 2 General Surgery, Cleveland Clinic Foundation, Cleveland, USA; 3 Gastroenterology, Cleveland Clinic Foundation, Cleveland, USA

**Keywords:** fundoplication, dyspepsia, epigastric pain syndrome, post-prandial distress, refractory gerd

## Abstract

Post-fundoplication dyspepsia is a common complication of gastric fundoplication surgeries. This can be attributable to the loss of fundal relaxation, decreased gastric accommodation, and/or alterations in gastric motility and sensitivity following fundoplication. The role of neuromodulators in the management of such symptoms is unknown. We retrospectively assessed the efficacy of neuromodulators such as tricyclic antidepressants, buspirone, and mirtazapine for the management of post-fundoplication dyspepsia.

## Introduction

Gastroesophageal reflux disease (GERD) is a common gastrointestinal disorder worldwide with an estimated global prevalence of 8%-33% [1,2]. Antireflux surgery such as fundoplication is at times performed in nonobese patients with symptoms of reflux esophagitis refractory to medical therapy with proton pump inhibitors (PPIs) or in patients with symptomatic large hiatal hernias [3-5] or in patients who are unable to tolerate chronic medical therapy [6,7]. Gastric fundoplication surgeries such as Nissen or Toupet procedures effectively reduce the reflux symptoms on short and prolonged follow-up periods [8,9]. However, the post-surgical course can be complicated by dyspeptic symptoms such as epigastric pain or pressure, bloating, early satiety, and difficulty in belching after surgery [9,10]. These symptoms may be attributed to a reduction in the gastric accommodation due to the loss in the fundic volume after surgery, alterations in gastric emptying, visceral hypersensitivity, or a combination of these factors after surgery [11,12].

Various treatment options are available for the management of functional dyspepsia (FD), but there is no US Food and Drug Administration (FDA)-approved therapy for FD or post-fundoplication surgery dyspepsia [13,14]. Neuromodulators such as low-dose tricyclic antidepressant, buspirone, and mirtazapine have shown promise in alleviating symptoms of FD [15-17]. However, the gastric anatomy is altered post-fundoplication; the proximal stomach is wrapped around the gastroesophageal junction compromising the gastric accommodation. The utility of neuromodulators in these patients is unexplored. At present, there are no studies that have evaluated the efficacy of neuromodulator therapies in this patient population.

The objective of the present study was to assess the efficacy of neuromodulators for the treatment of dyspeptic symptoms in patients with prior antireflux fundoplication surgery.

## Materials and methods

Study design

This was a single-center, retrospective, Institutional Review Board-approved study at the Cleveland Clinic.

Patient, definitions, and procedures

Consecutive patients with prior gastric fundoplication surgery from January 1, 2010, to December 31, 2020, were screened. Patients presenting with symptoms of dyspepsia diagnosed per the current Rome IV criteria for dyspepsia and started on mirtazapine, buspirone, or tricyclic antidepressant for these symptoms were eligible [18]. Per these criteria, symptoms of post-prandial fullness, early satiety, epigastric pain or epigastric burning, and unexplained after routine medical evaluation characterize dyspepsia. Patients were excluded if neuromodulator was prescribed for other disorders such as anxiety, depression, or musculoskeletal pain.

Dyspepsia was diagnosed based on the Rome IV criteria [18]. Patients presenting with meal-associated dyspeptic symptoms were classified as post-prandial distress syndrome (PDS), while those with abdominal pain or burning unrelated to meals were classified as epigastric pain syndrome (EPS). Patients with both the symptom complexes were classified as an overlap syndrome. For the diagnosis of FD, the symptoms should be active for at least three months prior to the date of diagnosis and have started at least six months prior to the diagnosis.

Study measurements

Individual patient details like age at the onset of dyspepsia, years since the fundoplication surgery, symptoms, weight loss, prior investigations such as gastric emptying studies, and high-resolution manometry were collected. Patients were diagnosed with rapid gastric emptying if there was less than 30% of gastric retention at one hour and with delayed gastric emptying if there were more than 10% of gastric contents at four hours. The manometry findings were interpreted based on Chicago classification v3.0 [19]. The type of medication started, the initial dose of the medications, the treatment response, and the associated adverse effects after initiation of the medication were noted.

Post-treatment initiation, the response was noted based on the patient’s subjective improvement on therapy, weight changes after initiation of the therapy, adverse effects of the therapy, and treatment continuation or discontinuation after follow-up. Patients were followed up till the resolution of symptoms or changes in the therapy.

## Results

Patient characteristics

Sixty-seven patients with prior fundoplication surgery were screened, nine patients with dyspeptic symptoms who were prescribed neuromodulators were included and the remaining 58 patients were excluded, as the neuromodulators were prescribed for indications other than dyspepsia. The median age of presentation with dyspepsia was 57 (18-81) years; the median time interval between the surgery and the onset of dyspepsia was 4.9 (0.6- 20.3) years (Table 1). Majority of the patients were females (7, 77%), and all the patients were Caucasian. Eight of these patients had undergone Nissen fundoplication, while one had undergone Toupet fundoplication. One patient developed dysphagia after the initial Nissen procedure, and the fundoplication was converted to a Toupet fundoplication. All the patients had prior upper endoscopy to rule out other causes of dyspepsia and were negative. Nuclear gastric emptying test was performed for six patients, and gastric emptying at four hours was normal in all of these patients. Pre-surgical manometry was available for four patients and normal in all patients. No patient in the cohort underwent manometry after surgery.

Treatment outcomes

Four patients presented with EPS, three presented with PDS, while two patients had mixed symptoms (Table 1). All the patients with PDS had significant symptom resolution after initiation of neuromodulator therapy. Two patients were started on buspirone therapy. One patient responded well to the buspirone alone and continued the medication. One patient with associated neuropathic cough was started on an amitriptyline in addition to buspirone and had significant improvement in all symptoms; he attributed his improvement to amitriptyline and discontinued the buspirone. One patient was started on amitriptyline and reported significant improvement in her post-prandial fullness and bloating after therapy initiation. 

**Table 1:** Clinical characteristics and treatment response of the individual patients *At the time of clinical presentation. ‡Reported increased somnolence and discontinued therapy. †Converted Nissen to Toupet fundoplication due to the development of severe dysphagia after the surgery. §Hypotensive lower esophageal sphincter pressure on post-surgery high-resolution manometry. EPS: epigastric pain syndrome, F: female, M: male; PDS: post-prandial distress syndrome.

Age* (years)	Time since surgery (years)	Fundoplication type	Symptoms	Functional dyspepsia subtype	Weight changes*	Intervention	Response	Total follow-up (years)
19/F‡	0.4	Nissen	Epigastric pain	EPS	None	Amitriptyline 10 mg	No improvement and patient could not tolerate the dose due to increased somnolence.	0.5
43/M	0.7	Nissen	Bloating, epigastric pain	EPS	None	Amitriptyline 10 mg	No Improvement in the symptoms.	0.5
62/F	3.5	Toupet	Epigastric pain	EPS	None	Amitriptyline 10 mg, Mirtazapine 15 mg	Significant improvement in the abdominal pain.	3
72/F	6.4	Nissen	Epigastric pain, nausea	EPS	Lost 12 pounds	Buspirone 10 mg	Improvement in the pain, nausea, and weight gain.	4
58/M	0.4	Nissen†	Bloating	PDS	5 pounds weight gain	Buspirone 10 mg, Amitriptyline 10 mg	Also had functional cough Improvement with amitriptyline. Stopped buspirone as it did not significantly improve the PDS.	3
51/F§	4.9	Nissen	Bloating, nausea	PDS	None	Amitriptyline 10 mg	Symptoms of bloating and nausea improved and continued therapy.	4
81/F	20.8	Nissen	Epigastric fullness, nausea	PDS	Lost 25 pounds	Buspirone 10 mg, Mirtazapine 15mg	Significant improvement and weight gain of 17 pounds.	4
36/F	7	Nissen	Epigastric pain, bloating	Mixed	None	Buspirone 5 mg	No response. Stopped medications.	2
58/F	0.4	Nissen	Epigastric pain, bloating	Mixed	None	Buspirone 10 mg	Some improvement and continued to take the medication. Also associated improvement in anxiety.	5

Of the four patients who presented with symptoms of EPS, two patients reported improvement in their symptoms after neuromodulator therapy initiation and both continued taking the medication. One patient was started on a combination of amitriptyline and mirtazapine therapy, while another was started on buspirone. Both had significant improvement in epigastric pain. One patient was started on amitriptyline therapy alone but had no improvement and discontinued the therapy soon after.

Two patients had mixed EPS/PDS symptoms. One patient was started on combined buspirone and mirtazapine therapy for the weight loss. She had significant symptom improvement with the therapy, regained her lost weight, and continued both medications long term. The other patient did not have any significant response to buspirone and discontinued the medication.

Drug-related adverse events

One patient had intolerable symptoms related to the neuromodulator use. This patient presented with EPS and suffered from drug-related drowsiness and somnolence after the initiation of amitriptyline therapy. Her dose was initially halved but had to be discontinued as the somnolence did not improve despite the dose reduction. 

## Discussion

Dyspepsia is common but is typically reported as a transient complication of antireflux surgeries [20,21]. Persistent dyspeptic symptoms following antireflux surgery may significantly affect the quality of life. To date, there exists minimal literature regarding the management of post-fundoplication dyspepsia. In the present retrospective series, treatment with neuromodulators was associated with symptom resolution in all patients with PDS, two of the four patients with EPS, and one of two patients with mixed EPS/PDS. These medications were well-tolerated by all but one patient. To the best of our knowledge, this is the first study that has evaluated the role of neuromodulator medications in patients with chronic dyspepsia following surgical fundoplication. 

The pathophysiological mechanisms underlying FD are heterogeneous. Theoretic causes include impaired gastric accommodation, altered gastric emptying, and increased gastric hypersensitivity [13,22-24]. Previous studies have demonstrated that patients presenting with post-fundoplication dyspepsia have significantly reduced gastric accommodation compared to controls, presumably due to surgical alteration of the gastric fundus [11,12].

Buspirone, a serotonin-1A (5-HT1A) receptor agonist medication, has shown to improve gastric accommodation and relieve the symptoms of dyspepsia, especially PDS [17,25]. In our study, two of three PDS patients who started buspirone had significant improvement in symptoms. Additionally, one patient with EPS reported significant subjective improvement and weight gain after initiation of buspirone. These findings would support the hypothesis of impaired gastric accommodation leading to symptoms in patients with prior fundoplication. Amitriptyline has proven efficacy in managing EPS symptoms in patients with FD [15,16]. Of the three patients with epigastric pain who started amitriptyline, one reported significant improvement in symptoms. One patient did not continue taking amitriptyline due to somnolence. Mirtazapine, a 5-HT1 agonist and 5-HT2 and 5-HT3 antagonist, has been shown to be effective in FD patients with concomitant weight loss [26,27]. In the present study, it was used in two patients. One of the patients reported significant weight gain after initiation of the therapy, while the other patient had no response. Our study has limitations. It was a retrospective study with a small number of patients, which could lead to a selection bias. Also, assessment of the treatment efficacy was subjective based on the patient’s symptomatic response and willingness to continue the medications. Nevertheless, there was objective weight gain in two of the patients who reported prior weight loss. Further prospective studies should be considered to thoroughly assess the impact of neuromodulator therapies in the treatment of dyspepsia in post-fundoplication patients. The various other modalities for the management of dyspepsia such as prokinetic agents (domperidone), herbal preparations of STW5 (a combination of nine herbal extracts), peppermint, and caraway oil preparations have been shown to be effective in the management of FD [16,28-30]. The role of these modalities in patients with prior fundoplication surgery needs to be explored in detail.

## Conclusions

In conclusion, we found that neuromodulator therapies can be an effective therapy for the management of post-fundoplication dyspepsia especially in patients presenting with post-prandial distress syndrome.
